# Expression of 9-*O*-Acetylated Sialic Acid in HPV+ Oral Squamous Cell Carcinoma Cells

**DOI:** 10.3390/life15040663

**Published:** 2025-04-17

**Authors:** Hugo Sánchez-Martínez, Victoria Jiménez-Castillo, Daniela Illescas-Barbosa, Beatriz Xochitl Ávila-Curiel, María Teresa Hernández-Huerta, Risk Díaz-Castillejos, Rafael Torres-Rosas, Edgar Zenteno, Mohamed Alí Pereyra-Morales, Carlos Josué Solórzano-Mata

**Affiliations:** 1 Faculty of Dentistry, Universidad Autónoma Benito Juárez de Oaxaca, Oaxaca 68120, Mexico; drhugosanchezoaxaca@gmail.com (H.S.-M.); tomy_dib@hotmail.com (D.I.-B.); beatrizxochitla@gmail.com (B.X.Á.-C.); riskdc@hotmail.com (R.D.-C.); rtorres.cat@uabjo.mx (R.T.-R.); 2 Faculty of Medicine and Surgery, Universidad Autónoma Benito Juárez de Oaxaca, Oaxaca 68120, Mexico; jimcv_28@hotmail.com; 3 SECIHTI-Faculty of Medicine and Surgery, Universidad Autónoma Benito Juárez de Oaxaca, Oaxaca 68120, Mexico; mthernandez@conahcyt.mx; 4 Departament of Biochemistry, Faculty of Medicine, Universidad Nacional Autónoma de México, Ciudad de México 04510, Mexico; ezenteno@unam.mx (E.Z.); ali@bq.unam.mx (M.A.P.-M.)

**Keywords:** OSCC, Neu5,9Ac_2_, Neu5Ac, *Macrobrachium rosenbergii* lectin, SCC-152, HaCaT, prognostic tools, glycosylation

## Abstract

Oral squamous cell carcinoma (OSCC) is a common type of head and neck malignancy that represents a significant global health issue. Sialylations are common events in tumor transformation, proliferation, metastasis, and immune evasion. Modifications in sialylation can be detected by lectins, whose changes in OSCC have been related to grade, invasion, and metastasis. The presence of 9-*O*-acetylated sialic acid (Neu5,9Ac_2_) in OSCC cells and its potential expression, modification, and role are unknown. This study aimed to analyze the expression of Neu5,9Ac_2_ using the *Macrobrachium rosenbergii* lectin (MrL) that recognizes this sialic acid (Neu5Ac) residue and also compare its effect on the SCC-152 cell line (CRL-3240, ATCC) and immortalized keratinocytes (HaCaT) as a control. We observed by immunocytochemistry that SCC-152 cells expressed more Neu5,9Ac_2_ compared to HaCaT cells; the specificity of MrL was confirmed after the sialidase treatment of cells in which the loss of lectin’s recognition of Neu5,9Ac_2_ was observed. The electrophoretic profile was similar between both cell line types; however, the Western blot showed differences in the glycoprotein patterns recognized by lectin for each cell type. MrL increased the proliferation of SCC-152 cells, as well as the integrity and morphology of the colonies. Therefore, our results suggest that Neu5,9Ac_2_ glycosylated receptors could be involved in the survival and proliferation of OSCC cells, which offers a promising avenue for developing diagnostic and prognostic tools (tumor markers) against oral squamous cell carcinoma in the future.

## 1. Introduction

Oral squamous cell carcinoma (OSSC) is the main malignant tumor within the group of head and neck tumors, commonly affecting older people with a history of tobacco and alcohol use [1,2,3]. The overall survival rate observed at 5 years is 59%, and 45.7% of patients presented with postoperative recurrence or metastasis [4]. There is a distinctive subgroup of patients with Human Papillomavirus (HPV) infection, affecting young individuals with risky sexual behaviors and unrelated to tobacco and alcohol use [5]. Affected individuals are mainly male, with the involvement of the oropharynx and oral cavity. Oropharyngeal carcinoma is associated with HPV in 70–90% of cases; however, the association of OSCC with HPV varies depending on the population studied. For example, in India, prevalences of 16.7% [6] and 25.6% [7] have been reported, in Brazil, 26.7% [8], in the USA, 31% [9], and in Mexico, 42% [10]. Additionally, it has been documented that in HPV-positive OSCC patients, the median survival time at one year (95%), 3 years (78.5%), and 5 years (38.5%) was significantly higher than that of the HPV-negative 1-year (78.6%), 3-year (45.2%) and 5-year (38.5%) OSCC patients [11]. These data suggest the role of HPV in carcinogenesis; what is clear is that HPV infection increases the risk of cancer at any site of the oral cavity [12] and that there is an increase in the number of HPV-related cases [13].

High-risk HPV types, such as genotypes 16 and 18, participate in oncogenesis through the production of viral oncoproteins [14]. For example, E6, when interacting with E6P (ubiquitin ligase E6AP), binds to and cleaves p53, causing an increase in the number of mutations and alterations in DNA replication [15]. E6 also inhibits Bax [16] and Bak [17], which negatively impact apoptosis. Additionally, this oncoprotein increases IAP-2, generating a positive effect on cell proliferation. Meanwhile, E7 inactivates the retinoblastoma protein (pRB) [18] and cyclin-dependent kinase inhibitor (p21) [19], which together promote cell survival. Furthermore, E7 activates the protein kinase B or Akt pathway (PKB/Akt), allowing the inhibition of apoptosis [20,21]. In addition, various target molecules are affected by HPV and participate in the cellular transformation process; however, other types of molecular processes are affected, including alterations in glycosylation and its functional impact at the cellular level.

Glycosylation is a co-translational and post-translational modification in which a series of carbohydrates are added to proteins [22], lipids [23], and nucleic acids [24]. Glycosylation is highly relevant because it is involved in various cellular processes that are altered in cancer, such as intercellular adhesion [25], proliferation [26], migration [27], signaling [28], and apoptosis [29].

**Figure 1:**
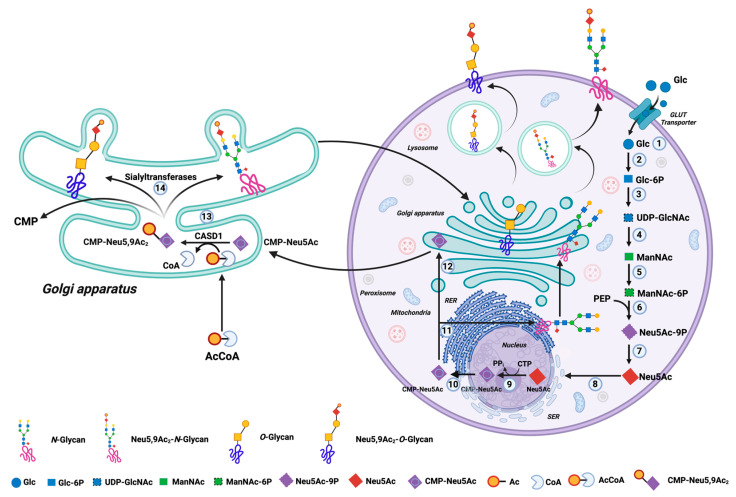
The biosynthesis of Neu5Ac in mammalian cells and the acetylation of sialic acid at carbon 9. The synthesis of sialic acid in vertebrate cells begins in the cytosolic compartment. (1) Once glucose (Glc) is located intracellularly at this site, (2) it is recognized by hexokinase to produce Glucose-6-phosphate (Glc-6P), (3) and then this product is metabolized by the hexosamine pathway to obtain UDP-N-Acetyl-D-glucosamine (UDP-GlcNAc). (4) Now, by the enzymatic action of UDP-GlcNAc-2-epimerase/ManNAc kinase on the previous metabolite in the presence of a water molecule, N-Acetylmonosamine (ManNAc-) is obtained. (5) ManNAc is recognized by UDP-GlcNAc 2-epimerase/ManNAc kinase and in the presence of ATP, N-Acetyl-monosmaine-6-phosphate (ManNAc-6-phosphate) is produced, and (6) together with Phosphoenolpyruvate (PEP), both substrates are recognized by the enzyme Neu5Ac-9-phosphate synthase to obtain N-Acetylneuraminic acid 9-phosphate (Neu5A-9-phosphate). (7) Subsequently, the catalytic activity of the Neu5Ac-phosphate phosphatase on Neu5A-9-phosphate plus a water molecule allows the production of sialic acid (Neu5Ac). (8) Neu5Ac is then transported into the nucleus (9) to combine with cytidine monophosphate (CMP) from cytidine triphosphate (CTP), thus obtaining the active form of sialic acid: CMP-Neu5Ac. Once CMP-Neu5Ac is obtained, it is sent to the (10) cytoplasm and transported to (11) the rough endoplasmic reticulum (RER) for early *N*-glycan synthesis or (12) to the Golgi apparatus to participate as a substrate in the terminal phase of *N*-glycosylation and *O*-glycosylation. Finally, (13) acetyl groups, donated from acetyl-CoA (AcCoA), are transferred for CMP-Neu5Ac by the action of the enzyme N-acetylneuraminate (7)-9-*O*-acetyltransferase (CASD1) to obtain CMP-N-Acetyl-beta-neuraminate (CMP-Neu5,9Ac_2_), (14) a molecule that is used in the Golgi apparatus as a substrate for the addition of terminal 9-*O*-acetylated sialic acid (Neu5,9Ac_2_) in *N*-glycan and *O*-glycan. The image is created by the authors using http://biorender.com/ (accessed on 22 March 2025).

The two main types of glycosylation are *N*-glycosylation and *O*-glycosylation. *N*-glycosylation is a co-translational modification that begins in the rough endoplasmic reticulum (RER) and continues in the Golgi apparatus. The addition of carbohydrates starts at the consensus sequence Asn-X-Ser/Thr (asparagine—any amino acid except Proline, Serine or Threonine) of proteins to form three general types of N-glycans: oligomannose, complex, and hybrid. *N*-glycans are localized to cell membranes or secreted [30]. *O*-glycosylation is a post-translational modification in the Golgi apparatus on Ser/Thr (Serine/Threonine) amino acids. The main carbohydrate that begins the oligosaccharide chain is N-Acetylgalactosamine (GalNAc) with its subsequent elongation by different cores. Once the *O*-glycans are synthesized, they are transferred to the cell membranes and/or are secreted into the extracellular space [31]. Finally, in the terminal region of both types of glycans, sialic acid (Neu5Ac) can be incorporated in a process known as sialylation (Figure 1) [31].

Neu5Ac is a 9-carbon carbohydrate with a negative charge at physiological pH, and it is often located in the terminal portion of *N*-glycans, *O*-glycans, and glycosphingolipids. Neu5Ac is linked via a glycosidic bond between linear oligosaccharides and underlying branches in glycoproteins and glycolipids, usually located in the outermost region of the oligosaccharide chain in α2-3- or α2-6- bonds with galactose or in α2-6 bond with N-Acetylgalactosamine (GalNAc) or N-Acetylglucosamine (GlcNAc) [32,33,34].

The synthesis of Neu5Ac in vertebrate cells begins in the cytosolic compartment. Once glucose (Glc) is located intracellularly at this site, it is recognized by hexokinase to produce Glucose-6-phosphate (Glc-6P), and then this product is metabolized by the hexosamine pathway to obtain UDP-N-Acetyl-D-glucosamine (UDP-GlcNAc). Now, by the enzymatic action of UDP-GlcNAc-2-epimerase/ManNAc kinase on the previous metabolite in the presence of a water molecule, N-Acetylmonosamine (ManNAc) is obtained. ManNAc is recognized by UDP-GlcNAc 2-epimerase/ManNAc kinase, and in the presence of ATP, N-Acetyl-monosmaine-6-phosphate (ManNAc-6-phosphate) is produced, and together with Phosphoenolpyruvate (PEP), both substrates are recognized by the enzyme Neu5Ac-9-phosphate synthase to obtain N-Acetylneuraminic acid 9-phosphate (Neu5A-9-phosphate). Subsequently, the catalytic activity of the Neu5Ac-phosphate phosphatase on Neu5A-9-phosphate plus a water molecule allows the production of Neu5Ac (Figure 1) [35].

Neu5Ac is then transported into the nucleus to combine with cytidine monophosphate (CMP) from cytidine triphosphate (CTP), thus obtaining the active form of Neu5Ac: CMP-Neu5Ac. Once CMP-Neu5Ac is obtained, it is sent to the cytoplasm and is transported to the rough endoplasmic reticulum (RER) for early *N*-glycan synthesis in the Golgi apparatus to participate as a substrate in the terminal phase of *N*-glycosylation and *O*-glycosylation. Finally, acetyl groups, donated from acetyl-CoA (AcCoA), are transferred to CMP-Neu5Ac by the action of the enzyme N-acetylneuraminate (7)-9-*O*-acetyltransferase (CASD1) to obtain CMP-N-Acetyl-beta-neuraminate (CMP-Neu5,9Ac_2_), a molecule that is used in the Golgi apparatus as a substrate for the addition of terminal 9-*O*-acetylated sialic acid (Neu5,9Ac_2_) in *N*-glycan and *O*-glycan [35] (Figure 1).

During cellular transformation, sialyltransferase activity in the Golgi apparatus is increased [36], with the consequent hypersialylation that comprises 40–60% of the surface of tumor cells [37]. Cancer cells express a greater amount of highly sialylated tumor-associated glycosylated antigens that can be released into the extracellular space [38]. The presence of Neu5Ac-α2-6- is low or absent in various healthy tissues [39]. However, its overexpression is associated with a metastatic phenotype, for example, colon carcinoma [40,41], hepatocarcinoma [42,43], breast cancer [44], and cervical cancer [45,46]. In this regard, total Neu5Ac levels in unstimulated saliva are found in a higher concentration in patients with OSCC compared to individuals with oral potentially malignant disorders (OPMDs) and healthy people [47,48,49]. In addition, a significant increase in salivary and serum Neu5Ac has been observed in patients with OSCC compared to healthy individuals [50]. Also, salivary and serum Neu5Ac levels have been correlated with the histopathological grade of OSCC [51]. These data together suggest that the products obtained from sialylation throughout the tumor transformation could be used as a potential tumor marker for OSCC [52,53,54,55].

Lectins are a tool used to study glycosylation, which is the attachment of sugar residues to proteins or lipids. Lectins are non-immune proteins that specifically recognize carbohydrates. Plant lectins and animals are used to define glycophenotypes in cells and tissues [56]. In the case of sialylation, lectins allow the recognition of Neu5Ac in α2-3 or α2-6 bonds with galactose, through α2-6 bonds with GalNAc or GlcNAc, and through α2-8 bonds with other Neu5Ac that form polysialic acids [33]. One of the most common changes in glycoconjugates during malignant transformation is the increase in the size of oligosaccharides, which results in branched sites for Neu5Ac incorporation [49]. Classically, two plant lectins have been used for its study, the Neu5Acα2-3-specific lectin from *Maackia amurensis* (MAA) and the Neu5Acα2-6-recognizing lectin from *Sambucus nigra* (SNA). For example, in OSCC, Neu5Ac in α2-3 and α2-6 junctions detected, respectively, by the lectins of MAA and SNA, is found to be increased in cancerous tissues compared to normal adjacent tissue [52,53,57].

A common Neu5Ac modification is the addition of one or more *O*-acetyl esters to the hydroxyl groups of Neu5Ac residues, yielding different *O*-Ac-Sia forms associated with different glycoconjugates [58]. Neu5,9Ac_2_ has been detected in several malignant tumors, including acute lymphoblastic leukemia (ALL) [59], melanoma [60], basal cell carcinoma [61], and breast cancer [62]. However, it is unknown how it is expressed in OSCC.

A 20 kDa glycoprotein with lectin activity (MrL) has been purified from the hemolymph of adult *Macrobrachium rosenbergii* specimens. This protein consists of two 9.6 kDa subunits and specifically binds to Neu5,9Ac_2_ in glycopeptides within the saccharide sequence Neu5,9Ac2α2,6Galβ1,3GalNAc [63]. MrL exhibits a high affinity for *O*-acetylation at carbon 9, a modification previously documented in Neu5Ac [64]. Notably, MrL has been employed to identify patients with T-cell ALL, highlighting its potential as a valuable tool for studying Neu5,9Ac_2_ in other neoplastic diseases [65].

Thus, this study aimed to analyze the expression of Neu5,9Ac_2_ in glycoproteins using MrL and evaluate its effects on the SCC-152 cell line. SCC-152 cells are a grade 2 squamous cell carcinoma cell line isolated from the hypopharynx (recurrent), originating from a tumor at the base of the tongue. These cells are HPV16-positive and have been instrumental in the molecular study of oral cancer [66,67,68,69]. For the above, it is essential to characterize both structural and functional molecules for their potential use as tumor biomarkers [70] or therapeutic targets [71]. Among them, carbohydrates play a significant role [72,73].

## 2. Materials and Methods

### 2.1. Cell Culture

The SCC cell line UPC:SCC-152 (ATCC CRL-3240, University Bolulevard Manassas, VA, USA) was obtained from the American Type Culture Collection (ATCC). These are HPV-positive cells with a predominantly colony-like growth pattern. The cells were cultured in 25 cm^2^ culture bottles (Corning, Inc., Corning, NY, USA, 3055) in Earle’s Minimum Essential Medium (EMEM) (Caisson, Logan, UT, USA), supplemented with 10% *v*/*v* Fetal Bovine Serum (FBS) (Biowest, Mexico City, Mexico) and penicillin/streptomycin (100 U/mL) (Caisson, Logan, UT, USA), at 37 °C in a humidified atmosphere with 5% CO_2_ and 95% air. The cells were periodically evaluated for morphology and quantity under an inverted microscope (Zeiss, Oberkochen, Germany).

HaCaT is a spontaneously immortalized, non-tumorigenic human cutaneous keratinocyte line with a monolayer growth pattern. Cells were cultured in 25 cm^2^ culture bottles (Corning, Inc., Corning, NY, USA, 3055) in high glucose Dulbecco’s Modified Eagle Medium (DMEM) (Gibco, Stanley Rd., Grand Island, NY, USA) supplemented with L-Glutamine (4 mM) (Sigma, St Louis, MO, USA), Sodium Pyruvate (1 mM) (Sigma, St Louis, MO, USA), FBS (Biowest, Mexico) at 10% *v*/*v*, and Penicillin/Streptomycin (100 U/mL) (Caisson, Logan, UT, USA). Cells were cultured under the conditions described above and used as the line control.

### 2.2. Macrobrachium Rosenbergii Lectin

MrL was purified by affinity chromatography using stroma from rat erythrocytes [60]. It was donated by Dr. María Concepción Agundis-Mata of the Department of Biochemistry of the Faculty of Medicine of the Universidad Nacional Autónoma de México.

### 2.3. Immunocytochemistry

SCC-152 and HaCaT cells reached 80% confluence and were detached with Trypsin-EDTA (Ethylenediaminetetraacetic acid) at 0.05% (Caisson, Smithfield, UT, USA), and 1 × 10^5^ cells per well were plated on a Lab-Tek chamber slide w/cover (glass sterile) with 8 wells (Thermo Fisher Scientific, Rochester, NY, USA) with the respective culture medium. After 24 h of culture, the supernatant was removed, the cells were washed with Phosphate-Buffered Saline (PBS) [(50 mM sodium phosphate (J. T. Baker, Phillipsburg, NJ, USA), 0.15 M sodium chloride (NaCl, J. T. Baker, USA)] 1x pH 7.4 and fixed with Paraformaldehyde (PFA) (Sigma, St Louis, Mo, USA) at 4% in PBS 1x pH 7.4 for 20 min at room temperature in the dark; then, the slide was washed with PBS 1x pH 7.4. Nonspecific binding sites were blocked with 2% IgG-free bovine serum albumin (BSA) (ThermoScientific, Meridian Rd, Rockford, IL, USA) for 30 min. Then, the cells were permeabilized with Triton X-100 (Sigma, St Louis, MO, USA) at 0.2% in PBS 1x pH 7.4 for 10 min. Next, the avidin–biotin blocking system (Invitrogen by Thermo Fisher Scientific, Waltman, MA, USA) was used for 10 min at room temperature, and the slides were washed with PBS 1x pH 7.4 and PBS-Ca^++^ 1x pH 7.4 and immediately incubated with MrL-biotin 1:100 at 4 °C overnight. The next day, the slides were washed with PBS 1x pH 7.4, incubated with Streptavidin-Alexa594 (Life Technologies, Eugene, OR, USA) for 30 min at room temperature in the dark and washed with PBS 1x pH 7.4. Finally, the slide was mounted with FluoshieldTM with 4′,6-diamidino-2-fenilindol (DAPI) (Sigma, St Louis, MO, USA). The slides were observed under a LEICA DM2000 fluorescence microscope (Wetzlar, Germany), and the images were processed in Fiji/ImageJ software Version 1.54p.

### 2.4. Sialidase Treatment of Cells

The cells were fixed with 4% PFA in PBS as previously described, and they were treated with sialidase (a sialic acid hydrolysis assay), as reported by Mayoral et al., 2008 [74]. In brief, the fixed slides were incubated with *Clostridium perfingers* sialidase (Sigma, St Louis, MO, USA) in PBS 0.1 I.U. at different time intervals (1, 2, 3, and 4 h) at 37 °C in a humid chamber. The cells were then processed, as mentioned in the Immunocytochemistry Section.

### 2.5. Cell Lysate

SCC-152 and HaCaT cells were washed with PBS 1x pH 7.4 and incubated with Versene Solution [(3.04 grs Tris base (Madison, WI, USA), 8 grs NaCl (J. T. Baker, USA), 0.4 grs KCl (J. T. Baker, USA), EDTA (Sigma, St Louis, MO, USA)] for 10 min at 37 °C. Cells were recovered with a cell scraper (Falcon Cell Scraper, Corning, Reynosa, Mexico) and centrifuged at 1500 RPM for 10 min at 4 °C. The pellet was resuspended in lysis buffer [50 mM Tris-HCl (Madison, WI, USA), pH 8, 150 mM NaCl, 0.1% sodium dodecyl sulfate (SDS) (Madison, WI, USA), 1 mM EDTA (Sigma, St Louis, MO, USA), 1% NP40 (Sigma, St Louis, MO, USA), Sodium fluoride (Sigma, St Louis, MO, USA), β-glycerophosphate (Sigma, St Louis, MO, USA), sodium orthovanadate (Sigma St Louis, MO, USA), O-Phospho-L-Serine (Sigma, St Louis, MO, USA), sodium tetrabasic pyrophosphate (Sigma, St Louis, MO, USA), and SigmafastTM Protease Inhibitor Tablets (Sigma, St Louis, MO, USA)]. The suspension was vortexed for 1 min, incubated at 4 °C for 15 min, and then centrifuged at 12,000 RPM to remove cell debris for 30 min at 4 °C. Finally, the supernatant was aliquoted and stored at −20 °C until use.

### 2.6. Protein Quantification

Proteins were quantified using the bicinchoninic acid (BCA) method with the PierceTM BCA Protein Assay Kit (Rockford, IL, USA, Thermo Scientific) and following the protocol recommended by the manufacturer.

### 2.7. One-Dimensional Electrophoresis and Western Blot

Cell lysates were processed by electrophoresis and mixed with Laemmli buffer 1:5 (Sigma). Proteins (30 mg) were separated on a 10% SDS-polyacrylamide gel electrophoresis (SDS-PAGE) at a constant of 40 mA and stained with Coomassie Blue or transferred to nitrocellulose membranes (Rockford, IL, USA, ThermoSientific). To do this, the gel was incubated in transfer buffer [25 mM Tris base (Madison, WI, USA), 192 mM glycine (Madison, WI, USA), 20% methanol (J. T. Baker)] at a pH of 8.3 for 30 min.

Protein transfer to nitrocellulose membrane was performed in an ECL Semi Dry Blotters apparatus (Amersham, Biosciencies, Piscataway, NJ, USA) with constant amperage at 65 mA for 1 h in transfer buffer. Transfer efficiency was verified with 5% Ponceau red (Sigma). The membrane was washed with distilled water and blocked with PBS-albumin 5% for one hour, then the avidin–biotin blocking system (Invitrogen by Thermo Fisher Scientific, Waltman, MA, USA) was used for 10 min each at room temperature and then the membrane was washed with PBS-Tween 0.02% (Sigma) and PBS-Ca^++^ 1x pH 7.4. The membrane was then incubated with MrL-Biotin in PBS-Ca^++^ 1x pH 7.4 (1:1000) overnight at 4 °C. The next day, the membrane was washed with PBS-Tween 0.02% and incubated with ExtrAvidin^®^-Peroxidase (Sigma, St Louis, MO, USA) 1:4000 for one hour at room temperature. Finally, it was revealed by chemiluminescence with AmershamTM Prime Luminol Enhancer Solution (Buckinghamshire, UK, GE Healthcare Amersham). The bands were analyzed by densitometry using Fiji/ImageJ software, and the Gel Analyzer 2010 software was used to obtain molecular weights.

### 2.8. Cellular Proliferation

The proliferation assay with MTT [(4,5-dimethyl-2-thiazolyl)-2,5-diphenium-2H-tetrazolium bromide] (SIGMA, Merck KGaA, Darmstadt, Germany) was performed as reported by Hernández-Maqueda et al., 2013 [75]. For this, 1.5 × 10^5^ cells were cultured in 96-well plates with culture medium supplemented with 10% FBS overnight at 37 °C with 5% CO_2_, and then the cells were deprived of culture medium containing FBS for 24 h. The next day, MrL was added at concentrations of 1, 2.5 and 5 μg/mL for 24, 48 and 72 h.

At the end of the incubation times, the cells were incubated with 0.5 mg/mL of MTT for 3 h at 37 °C after isopropanol was added to each well to dissolve the formazan salts, and the absorbance was measured at 570 nm using a microplate spectrophotometer (MultiskanTM FC, Thermo Fisher).

### 2.9. Colony Formation Assay

To evaluate morphological characteristics and colony formation, SCC-152 and HaCaT cells were cultured and stimulated with MrL under the conditions described above; 4.5 × 10^5^ cells per well were considered [76]. Morphological characteristics were observed under an inverted microscope at 40× (Zeiss, Oberkochen, Germany), and microphotographs were recorded at 24, 48, and 72 h.

### 2.10. Statistical Analysis

Data are representative of three independent experiments, and values are expressed in mean ± SEM. Statistical analysis was performed in GraphPad Prism (version 10.4.1) for Windows, GraphPad Software, Boston, MA, USA, using ANOVA followed by Tukey’s test.

## 3. Results

### 3.1. SCC-152 and HaCaT Cells Express Neu5,9Ac_2_

**Figure 2:**
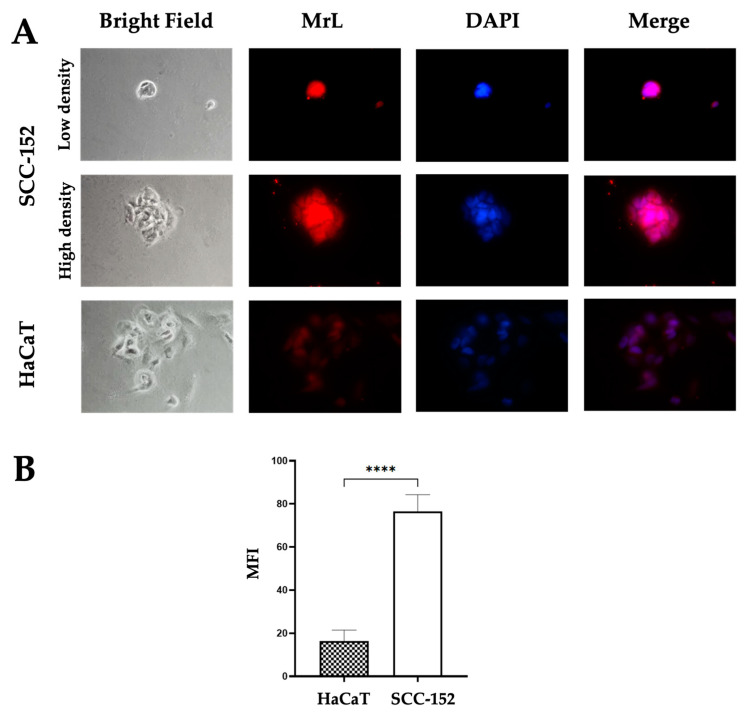
SCC-152 cells express higher levels of Neu5,9Ac_2_ compared to HaCaT cells. (**A**) SCC-152 and HaCaT cells were cultured in 10% FBS medium for 24 h and then fixed with 4% paraformaldehide. Indirect immunofluorescence was performed with MrL-biotin and Strptavidin-Alex594, as described in the Materials and Methods Section. Magnification: 40×. MrL (red) and nucleus (Blue). (**B**) The histogram displays the relative quantification of the MFI of the micrographs in Figure 2A by Fiji/ImageJ software (Version 1.54p). The results are the means ± SEM of 3 independent experiments. Statistical analysis is performed using one-way-ANOVA with using the Graphpad Prism Program (version 10.4.1). **** *p* < 0.0001. Abbreviations: MrL—*Macrobrachium rosenbergii* lectin; MFI—mean fluorescence intensity.

SCC-152 and HaCaT cell lines were recognized by MrL, with SCC-152 cells exhibiting a higher intensity of MrL localization compared to HaCaT cells (*p* < 0.0001) (Figure 2A,B). At the subcellular level, SCC-152 cells showed lectin localization in the nucleus, cytoplasm, and cell membrane, whereas HaCaT cells showed lower intensity and a more diffuse lectin distribution (Figure 2).

### 3.2. MrL Recognition Is Mediated by Neu5Ac

**Figure 3:**
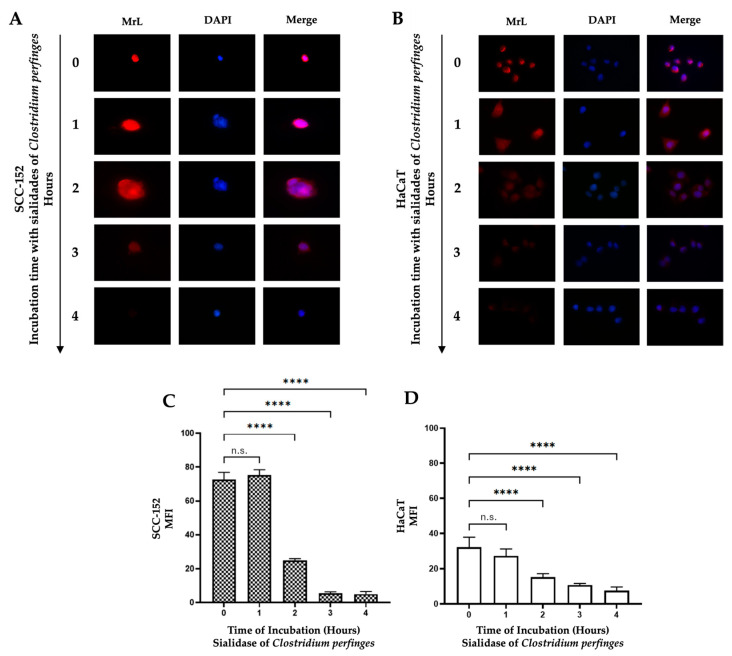
MrL recognition is mediated by sialic acid. (**A**) SCC-152 and (**B**) HaCaT cells were cultured as mentioned in the Materials and Methods Section and incubated with 0.1 IU of *Clostridium perfingers* sialidase at different time intervals (1–4 h) at 37 °C. Indirect immunofluorescence was performed with MrL-biotin and Strptavidin-Alex594 as described in the Materials and Methods Section. Magnification: 40×. MrL (red) and nucleus (Blue). The sialic acid hydrolysis assay shows a gradual decrease in MrL localization in both cell types over time. After 2 h of incubation, changes in fluorescence intensity are observed in the localization of the lectin compared to the control, with a very slight or almost non-existent intensity at 4 h in both cell types. (**C**,**D**) The histogram displays the relative quantification of the MFI of the micrographs in (**A**,**B**), respectively, by Fiji/ImageJ software (Version 1.54p). The results are the means ± SEM of 3 independent experiments. Statistical analysis is performed using one-way-ANOVA with using the Graphpad Prism Program (version 10.4.1). **** *p* < 0.0001. Abbreviations: MrL—*Macrobrachium rosenbergii* lectin; MFI—mean fluorescence intensity; n.s.—non-significant.

The Neu5Ac hydrolysis assay showed a gradual decrease in MrL localization in both cell types over time. As can be seen in Figure 3A,B, changes in the mean fluorescence intensity at the lectin location were observed after 2 h of incubation compared to the control, with statistically significant differences at 2, 3 and 4 h when compared to the control in SCC-152 and HaCaT cells (Figure 3C,D) (*p* < 0.0001). After 4 h of incubation with sialidase in both cell lines, MrL localization was minimal or nearly undetectable.

### 3.3. MrL Lectin Differentially Recognizes Glycoproteins in SCC-152 and HaCaT Cells

**Figure 4:**
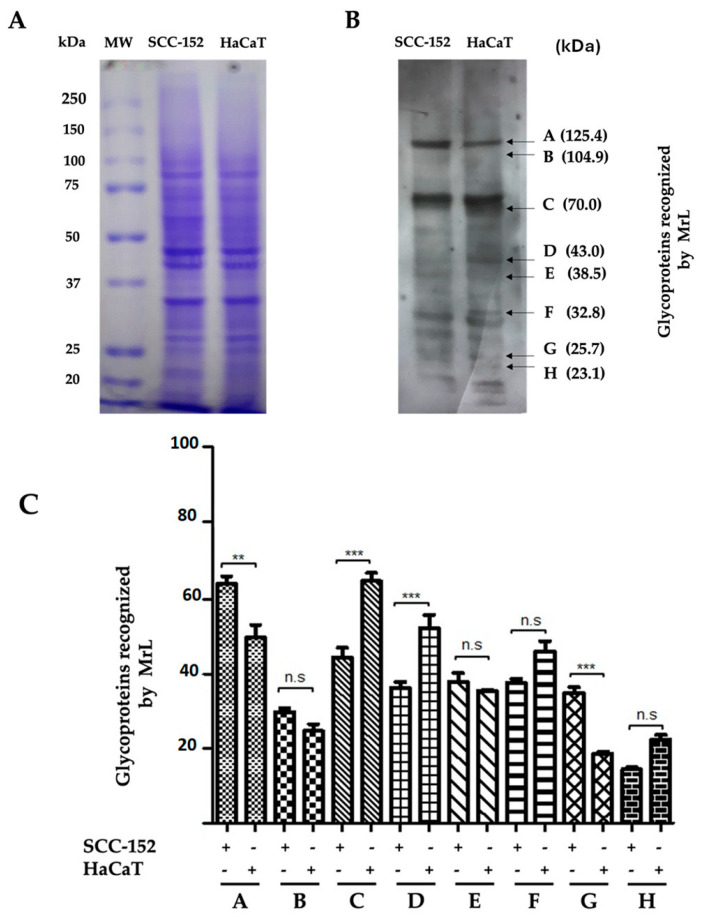
Glycoproteins recognized by MrL in SCC-152 and HaCaT cells. (**A**) Cell lysates from SCC−152 and HaCaT (30 μg) are run on 10% SDS-PAGE gels under reducing conditions. The gel is stained with Coomassie Blue to observe band resolution; however, no differences are found in the electrophoretic pattern between the two cell types. (**B**) A Western blot of SCC-152 and HaCaT cell lysates incubated with MrL reveals that the lectin recognizes different glycoproteins. (**C**) Glycoproteins A (125.4 kDa) and G (25.7 kDa) are overexpressed in SCC-152 cells compared to HaCaT cells, with statistically significant differences. In contrast, glycoproteins C (70 kDa) and D (43 kDa) show significant differences and, together with glycoproteins F (32.8 kDa) and H (23.1 kDa), show a decrease in their expression in SCC-152 cells compared to HaCaT cells. The results shown are representative of at least three independent experiments using different cell preparations. A densitometric analysis of the expression levels found for each glycoprotein is shown at the right, and the data represent the means ± SEM from at least three independent assays. Statistical analysis is performed by ANOVA followed by Tukey’s post-test. ** *p* < 0.01; *** *p* < 0.001; n.s., non-significant, compared to each condition.

The cell lysates of SCC-152 and HaCaT were processed by SDS-PAGE electrophoresis under reducing conditions. The gel was stained with Coomassie Blue, and the resolution of the bands was observed. As shown in Figure 4A, no differences were observed in the protein pattern in both cell types.

By Western blot using MrL in the lysates of SCC-152 and HaCaT cells, it was identified that the lectin recognized different glycoproteins ranging from 125 kDa to 23 kDa (Figure 4B). In the lysate of SCC-152 cells, it was identified that glycoproteins A (125.4 kDa) and G (25.7 kDa) are overexpressed compared to the lysate of HaCaT cells (** *p* < 0.01, *** *p* < 0.001, respectively). In contrast, glycoproteins C (70 kDa) and D (43 kDa) showed significant differences (*** *p* < 0.001) and, together with glycoproteins F (32.8 kDa) and H (23.1 kDa) (n.s., non-significant), showed a decrease in their expression in SCC-152 cells compared to HaCaT cells (Figure 4B,C).

### 3.4. MrL Induces Proliferation of SCC-152 Cells, While It Does Not Affect HaCaT Cells

**Figure 5:**
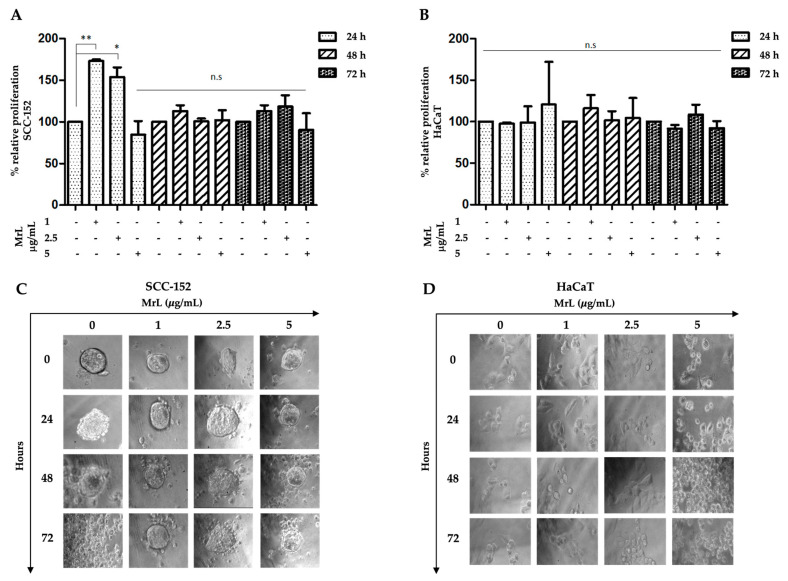
The effect of MrL on the proliferation and growth characteristics of SCC−152 and HaCaT cells. In this study, 1.5 × 105 cells are cultured in 96-well plates with culture medium supplemented with 10% FBS overnight, and then the cells are deprived of culture medium containing FBS for 24 h. The next day, MrL is added at concentrations of 1, 2.5 and 5 μg/mL for 24, 48 and 72 h. The proliferation assay with MTT is mentioned in the Materials and Methods Section. (**A**) The SCC-152 cell line shows a significant increase in cell proliferation at 24 h when incubated with MrL at concentrations of 1 and 2.5 μg/mL compared to the control. (**B**) In HaCaT cells, no effect on proliferation induced by MrL is observed at the concentrations and times used. To evaluate morphological characteristics and colony formation, SCC-152 and HaCaT cells (4.5 × 10^5^ cells per well) are cultured and stimulated with MrL under the conditions described above. Morphological characteristics are observed under an inverted microscope at 40×, and microphotographs are recorded at 24, 48, and 72 h. (**C**) MrL in SCC-152 cells causes a favorable effect on the preservation of colony integrity and morphology at all concentrations and times used. Colonies without lectin (control) show morphological alterations with poorly defined edges and an increase in the number of individual and dispersed cells. After 72 h, a total loss of colony integrity is observed, with a greater presence of dispersed cells. (**D**) MrL in HaCaT cells do not induce favorable changes in cell morphology or monolayer formation at any of the concentrations and incubation times used. MrL at a concentration of 5 μg/mL induces a negative effect on cell morphology after 24 h, while at 48 and 72 h, a loss of cell adhesion is observed compared to the control. The results represent the mean ± S.E.M. of 3 independent experiments. Statistical analysis is performed by ANOVA followed by Dunnett’s post-test. * *p* < 0.05; ** *p* < 0.01; n.s., non-significant.

The SCC-152 cell line showed a significant increase in cell proliferation at 24 h when incubated with MrL at concentrations of 1 (** *p* < 0.01) and 2.5 μg/mL (* *p* < 0.05) when compared to the control (Figure 5A). On the other hand, no effect was observed with any of the MrL concentrations at 48 and 72 h (n.s.) (Figure 5A). In HaCaT cells, no effect on proliferation was observed with MrL at 24, 48, and 72 h (Figure 5B).

### 3.5. MrL Maintains the Structural and Morphological Integrity of SCC-152 Cell Colonies

The results showed that the SCC-152 cell line incubated with MrL had a favorable effect on preserving the integrity and morphology of the colonies (Figure 5C). All concentrations used showed the previously mentioned effects; however, the most significant effects were identified with 1 and 2.5 μg/mL, even at 72 h. Colonies without lectin (control) presented morphological alterations with poorly defined edges and an increase in the number of individual cells. At 72 h, a total loss of colony integrity was observed, with a greater presence of dispersed cells (Figure 5C).

Regarding HaCaT cells, lectin did not induce favorable changes in cell morphology or the monolayer formation at any of the concentrations and incubation times used with lectin (Figure 5D). However, MrL at a concentration of 5 μg/mL induced a negative effect on cell morphology after 24 h, while at 48 and 72 h, there was a loss of cell adhesion compared to the control (Figure 5D).

## 4. Discussion

Several molecular changes have been associated with cellular transformation, including glycosylation. Changes in glycosylation are considered one of the main alterations in tumor transformation and progression. Consequently, cancer cells express a greater amount of highly sialylated tumor-associated glycosylated antigens that can be released into the extracellular space [38], which could be used as potential biomarkers for diagnosis and prognosis in oncology [77]. Currently, there are few tumor markers that have been shown to be sialylated glycoproteins and have demonstrated an impact at the clinical level, such as a-fetoprotein from liver cancer, CA-125 from ovary cancer, thyroglobulin from thyroid cancer, PSA from prostate cancer, and mucin from bladder cancer [77], hence the importance of identifying and characterizing this type of antigen due to its potential clinical application.

Neu5,9Ac_2_ is a sialic acid variant acetylated at carbon 9, which has been studied in certain leukemias and may also be expressed in other tumors. Several studies have documented hypersialylation during the carcinogenesis process in OSCC. Shah et al., 2008, reported a significant increase in total Neu5Ac levels and the high reactivity of *SNA* (Neu5Acα2,6Galβ1,4) and *MAA* (Neu5Acα2,3Galβ1,4) lectins, in addition to an increase in the activity of α2-3 and α2-6 sialyltransferases in OSCC tissues relative to their healthy counterparts [53,78,79]. On the other hand, the activity of α2-3 and α2-6 sialyltransferases and the TSA/TP (total Neu5Ac /total protein) ratio in saliva was higher in patients with OSCC with metastasis compared to patients without it [53,80,81]. Likewise, acetylation at carbon 9 of Neu5Ac has been shown to be present in different types of tumors, such as B-cell acute lymphoblastic leukemia (pre-B ALL), ALL in children [59], and basal cell carcinoma [61,82]. However, how it is expressed in OSCC, its function, and its application are unknown.

SCC-152 cells express a higher intensity of Neu5,9Ac_2_ compared to immortalized keratinocytes. However, Cavdarli et al., 2021, reported the localization of Ganglioside GD_2_ and its 9-*O*-acetylated form *O*AcGD_2_ in breast cancer cells (Hs 578T, SUM159PT, MDA-MB-231 GD3S+ and MCF7 GD3S+) on the cell surface and in “punctate” structures [83]. In this regard, gangliosides are glycosphingolipids that carry one or more Neu5Ac residues that modulate cell signaling, cause changes in the cell phenotype, and are affected in malignant transformation. Our data are consistent with the aforementioned study on the expression of Neu5,9Ac_2_ being present in cancer cells, particularly in our study on sialylated glycopeptides (Neu5,9Ac_2_), unlike GD_2_ glycosphingolipids, which are present in breast cancer cells [62,83].

Additionally, the interaction between MrL and SCC-152 cells or HaCaT via sialic acid was confirmed by the pretreatment of cells with *Clostridium perfringens* sialidase—an enzyme that catalyzes the removal of terminal Neu5Ac from a variety of glycoproteins and glycolipids with the production of free Neu5Ac [84,85]. This treatment led to the loss of the recognition of Neu5,9Ac_2_ by MrL in both cell types in immunocytochemical assays. Therefore, to confirm the presence of Neu5,9Ac_2_ in SCC-152 and HaCaT cells, it will be necessary to remove the 9-*O*-acetyl group using sialiac acid acetylesterase (SIAE) in the cells and continue with the incubation of MrL in cytochemical assays.

Moreover, studying the localization of MrL in OSCC tissues and their healthy counterparts is crucial to analyzing Neu5,9Ac_2_ expression and its variations. This could help establish its potential as a tumor marker in OSCC and correlate Neu5,9Ac_2_ levels with tumor differentiation and clinical staging.

From the proteins of the total lysate of the SCC-152 and HaCaT cell lines, a similar electrophoretic pattern was observed. However, in the Western blot assay, differences were observed between the pattern of glycoproteins recognized by MrL. In this regard, an increase in glycoproteins A and G was observed in SCC-152 compared to HaCaT. In addition, glycoproteins C, D, F, and H were more intense in HaCaT than in SCC-152. In this regard, the changes in expression levels between glycoproteins from both cell types are interesting because the identified differences could allow them to be used as potential tumor markers. Nevertheless, it is necessary to perform 2D electrophoresis assays that allow a greater characterization of the glycoproteins and protein sequencing to fully identify them. At this stage, one of the proteins characterized as carrying Neu5,9Ac_2_ is nuclein, a 100 kDa sialoprotein constitutively present on the surface of cancer cells in patients with pre-B ALL [86]. However, none of the glycoproteins identified in our study match the reported molecular weight of nuclein. The adequate characterization of glycoproteins that express this type of Neu5Ac is relevant. Chowdhury et al. (2008) report that the overexpression of Neu5,9Ac_2_ sialoglycoproteins can serve as a disease-associated antigen in lymphoblasts of children with ALL [87]. However, we provide an approximation of the glycoproteins that present this modification in OSCC cells and their healthy counterparts that must continue to be characterized.

To determine whether Neu5,9Ac_2_ plays a role in cell proliferation, MTT assays were performed at 24, 48, and 72 h in both cell types. Our results showed a significant increase in cell proliferation with MrL at 24 h with 1 and 2.5 μg/mL when compared to the control in SCC-152 cells; likewise, no effect was observed in HaCaT cells. The relationship between sialylation and cell proliferation in cancer is now well documented. For example, the downregulation of a2,6-sialyltransferase 1 (ST6Gal-I) promotes apoptosis and inhibits proliferation and invasion in cervical cancer cells [88]. Likewise, the downregulation of this enzyme in prostate cancer cells (PC-3 and DU145) significantly inhibits the proliferation, growth, migration, and invasion of these cells [89]. Our results on cell proliferation by Neu5,9Ac_2_ stimulation are in agreement with other studies; for example, the lectin *Achatinin-H (Achatina fulica)* specific for Neu5,9Ac_2_α2-6GalNAc induces proliferation with 0.1 μg at 48 h in peripheral blood mononuclear cells (PBMCs) derived from children with ALL [90]. On the other hand, the stimulation of tonsillar B cells treated with anti-IgM/IL-4 and anti-CD60b antibodies (specific for GD_3_-9-*O*-AcSA) induces cell proliferation; likewise, in tonsillar T lymphocytes, cell proliferation was induced when treated with PHA *(phytohemagglutinin)* and anti-CD60b [91]. The data suggest a correlation between Neu5,9Ac_2_ and cell proliferation, highlighting the need to investigate the signaling pathways underlying this effect. A limitation of our study is the lack of 9-*O*-acetyl group removal from Neu5Ac in the cells before the proliferation assay. This step would have provided a clearer confirmation of Neu5,9Ac_2_’s role in the observed biological effect.

We explored the effect of MrL on colonies in SCC-152. Our results showed that MrL induces a positive effect on the maintenance and integrity of colonies compared to the conditions previously exposed, but in HaCaT cells no result was observed regarding their growth pattern. However, a toxic effect of MrL was observed at 5 μg/mL. Our results suggest that the presence of Neu5,9Ac_2_ in OSCC cells is possibly related to intercellular adhesion by maintaining the integrity and morphology of colonies. The role of Neu5Ac in maintaining colony formation was reported in 1978 by Tonelli Q. and Meints RH. when they observed that treating bone marrow cells with *Vibrio cholerae* neuraminidase resulted in a reduction in the number of Colony Forming Units (CFUs) when transplanted into irradiated mice, suggesting a role for NeuAc in the implantation and development of CFUs [92]. Our results suggest that Neu5,9Ac_2_ may enhance the homotypic interaction of neoplastic cells, contributing to the formation of primary and metastatic tumors in patients. Meanwhile, Neu5Ac has been identified as a key ligand in interactions with various microorganisms [93]. However, the specific receptors that recognize Neu5,9Ac_2_ and function as adhesion molecules must be characterized.

## 5. Conclusions

In the present study, we identified the expression of Neu5,9Ac_2_ by MrL, which allowed us to identify its presence in cells of a SCC-152 cell line and its expression in the nuclear, cytoplasmic and plasma membrane compartments and glycoproteins, in addition to recognizing its effect on the proliferation and maintenance of colony integrity in vitro. Our findings highlight the critical role of Neu5,9Ac_2_ in glycoproteins as a potential biomarker for tumor diagnosis and prognosis. Additionally, its presence may help identify therapeutic targets capable of influencing tumor cell growth patterns and proliferation, opening the door to more precise and effective cancer treatments.

## Data Availability

The datasets used and/or analyzed during the current study are available from the corresponding author on reasonable request.

## Abbreviations

The following abbreviations are used in this manuscript:

AcCoa	Acetyl-CoA
*Achatinin-H*	*Achatina fulica*
Asn	Asparagine
ATCC	American Type Culture Collection
BCA	Bicinchoninic acid
BSA	Bovine serum albumin
CA-125	Cancer antigen 125
CASD1	N-acetylneuraminate (7)-9-*O*-acetyltransferase
CFUs	Colony Forming Units
CMP	Cytidine monophosphate
CTP	Cytidine triphosphate
DAPI	4′,6-diamidino-2-fenilindol
DMEM	Dulbecco’s Modified Eagle Medium
DU145	Prostate cancer cell line
E6	E6 oncoprotein
E6p	Ubiquitin ligase E6p
EDTA	Ethylenediaminetetraacetic acid
EMEM	Earle’s Minimum Essential Medium
FBS	Fetal Bovine Serum
GalNAc	N-Acetylgalactosamine
GD_2_	Ganglioside GD_2_
GD_3_-9-*O*-AcSA	Disialoganglioside GD_3_-*O*-Acetylate
Glc	Glucose
Glc-6P	Glucose-6-phosphate
GlcNAc	N-Acetylglucosamine
HaCaT	Non-tumorigenic human cutaneous keratinocyte
HPV	Human papillomavirus
MAA	*Maackia amurensis*
ManNAc	N-Acetylmonosamine
ManNAc-6-Phosphate	N-Acetylmonosamine-6-phosphate
MrL	*Macrobrachium rosenbergii* lectin
MTT	(4,5-dimethyl-2-thiazolyl)-2,5-diphenium-2H-tetrazolium bromide
NaCl	Sodium chloride
Neu5,9Ac	9-*O*-acetylated sialic acid
Neu5Ac	Sialic acid
Neu5Ac-9-phosphate	N-Acetylneuraminic acid-9-phosphate
OAcGD_2_	Ganglioside GD_2_-9-*O*-acetylated
OPMDs	Oral potentially malignant disorders
OSCC	Oral squamous cell carcinoma
PBMC	Peripheral blood mononuclear cell
PBS	Phosphate-Buffered Saline
PBS-Ca^++^	PBS-Calcio
PC-3	Prostate cancer cell line
PEP	Phosphoenolpyruvate
PFA	Paraformaldehyde
PHA	*Phytohemagglutinin*
pRB	Retinoblastoma protein
pre-B ALL	B-cell acute lymphoblastic leukemia
PSA	Prostate-specific antigen
RER	Rough endoplasmic reticulum
SCC	Squamous cell carcinoma
SCC-152	Squamous cell carcinoma cell line
SDS-PAGE	Sodium dodecyl-sulfate polyacrylamide gel electrophoresis
SER	Smooth endoplasmic reticulum
SIAE	Sialiac acid acetylesterase
SNA	*Sambucus nigra*
ST6Gal-I	α2,6-sialyltransferase I
Thr	Threonine
TP	Total protein
TSA	Total Neu5Ac
UDP-GlcNAc	UDP-N-Acetyl-D-glucosamine
